# Comparing Screening Abilities of the 33-Item Hypomania Checklist (HCL-33) and the 33-Item Hypomania Checklist External Assessment (HCL-33-EA) for the Detection of Bipolar Disorder

**DOI:** 10.3389/fpsyt.2021.518722

**Published:** 2021-06-22

**Authors:** Yuan-Yuan Wang, Yuan Feng, Meng Fang, Chengwei Guo, Gabor S. Ungvari, Brian J. Hall, Gang Wang, Yu-Tao Xiang

**Affiliations:** ^1^Division of Psychology, Faculty of Health and Life Sciences, De Montfort University, Leicester, United Kingdom; ^2^The National Clinical Research Center for Mental Disorders, Beijing Key Laboratory of Mental Disorders, Beijing Anding Hospital and the Advanced Innovation Center for Human Brain Protection, Capital Medical University, Beijing, China; ^3^University of Notre Dame Australia, Fremantle, WA, Australia; ^4^Division of Psychiatry, School of Medicine, University of Western Australia/Graylands Hospital, Perth, WA, Australia; ^5^New York University Shanghai, Shanghai, China; ^6^School of Global Public Health, New York University, New York, NY, United States; ^7^Unit of Psychiatry, Department of Public Health and Medicinal Administration, Institute of Translational Medicine, Faculty of Health Sciences, University of Macau, Macao, China; ^8^Centre for Cognitive and Brain Sciences, University of Macau, Macao, China; ^9^Institute of Advanced Studies in Humanities and Social Sciences, University of Macau, Macao, China

**Keywords:** bipolar disorder, sensitivity, specificity, HCL-33-EA, HCL-33

## Abstract

**Background:** Bipolar disorder (BD) is a severe psychiatric disorder that is often misdiagnosed and under-diagnosed in clinical settings. The 33-item Hypomania Checklist (HCL-33) is a newly developed self-administered scale for BD detection, while the 33-item Hypomania Checklist-external assessment (HCL-33-EA) is a version of the HCL-33 for external rating used by patient's carer (e.g., family member or friend). We aimed to compare the screening abilities between the HCL-33 and the HCL-33-EA, and evaluate the screening consistency between the two scales.

**Methods:** The data were collected from 269 patients with diagnosed BD (*n* = 84) or major depressive disorder (MDD) (*n* = 185). The sensitivity, specificity, positive predictive value (PPV), negative predictive value (NPV), and area under the curve (AUC) between the HCL-33 and the HCL-33-EA for BD were compared against clinician diagnosis as the gold standard.

**Results:** Using Youden's index, the optimal cut-off value of the HCL-33 is 20, while the corresponding figure for HCL-33-EA is 11. Using Youden's index, the HCL-33-EA showed a better performance than the HCL-33 (0.51 vs.0.41). The HCL-33-EA was more sensitive in correctly identifying BD patients from MDD patients than the HCL-33 (0.83 vs. 0.59), while the HCL-33 presented better specificity than the HCL-33-EA (0.82 vs. 0.68). There was significant screening consistency between the two scales (*p* < 0.001).

**Conclusions:** Both scales have acceptable psychometric properties in detection BD from MDD. Use of the two scales should be considered based on the assessment purpose in clinical research or daily practice (i.e., prefer sensitivity or specificity). Noticeably, the current sample size is insufficient and future studies are recommended to further evaluate the scales.

## Background

Bipolar disorder (BD) is a chronic and severe mood disorder comprising depressive and manic/hypomanic episodes (1). BD is frequently under-recognized, partly because patients are misdiagnosed as having major depressive disorder (MDD) during depressive episodes of BD (1–3). Moreover, BD patients usually do not report hypomanic episodes to clinicians, since patients may not experience impairment, and therefore do not consider hypomania as BD symptoms (2). Due to the commonly neglected hypomania, it is difficult to estimate the prevalence of misdiagnosed and underdiagnosed BD among those treated as MDD. Studies found that an average of 10 years was needed before accurate diagnoses of BD were established; in addition, around one-third of BD patients experienced at least once misdiagnosis (4, 5). The misdiagnosis of BD may have serious consequences, including high suicide risk (6) and low antidepressant treatment efficacy (7). Thus, it is crucial to distinguish BD accurately from other disorders, particularly MDD.

The Hypomania Checklists (HCL) are a series of widely used scales in detecting hypomanic symptoms and identifying BD, such as the 32-item Hypomania Checklist (HCL-32) (8), the 33-item Hypomania Checklist (HCL-33) (9), the 33-item Hypomania Checklist-external assessment (HCL-33-EA) (10), and their short versions (11, 12). The HCL-32 is a widely used patient-rated screening instrument for hypomanic symptoms with good psychometric properties in differentiating BD from MDD (8) and has been widely used in different countries (13–18). The HCL-33 is a recently developed questionnaire based on the extension of the HCL-32, which provides a more detailed assessment of hypomanic symptoms (9). The HCL-33-EA is the external assessment version of the HCL-33, which was designed to assess hypomanic symptoms by carers (such as spouses, parents, and friends) (10).

Several studies on the screening ability of the HCL-33 and the HCL-33-EA found satisfactory BD screening abilities of the two scales (9, 19). The consistency between the HCL-33 and the HCL-33-EA has been evaluated in a sample of Polish adults, which showed sufficient consistency between them (10). However, comparison between the HCL-33 and the HCL-33-EA for the detection of BD in patients with MDD and BD has not been conducted in China. The optimal cut-off value of the HCL-33 for distinguish BD from MDD is 15 in China (9), while the corresponding values of HCL-33-EA were not reported (10, 19). This is a critical gap in the literature, since there are an estimated 1.54 million people in China with BD (20). We aimed to compare the psychometric properties of the HCL-33 and the HCL-33-EA, including scale reliability, sensitivity, specificity, positive predictive value (PPV), negative predictive value (NPV), and area under the curve (AUC). In addition, we also measured the consistency between the HCL-33 and the HCL-33-EA, and aimed to provide optimal cut-off values of the HCL-33 and HCL-33-EA for distinguishing of BD from MDD in Chinese patients.

## Method

### Participants and Site

Following previous studies on psychometric properties of the HCL scales (11, 21–23), 269 inpatients and the same number of their carers were consecutively recruited between October, 2016 and January, 2019 in a major tertiary psychiatric hospital in Beijing, China. The patients were included if they were (1) adult patients diagnosed as MDD or BD depressive episode by two psychiatrists using the Mini-International Neuropsychiatric Interview Version 5.0 (24, 25) according to the International Classification of Diseases (ICD-10) (26), which was confirmed by a review of medical records; (2) could understand the contents of the interview. Patients with MDD or BD secondary to major medical conditions were excluded. All patients and their carer provided written consent and the Ethics Committee of Beijing Anding Hospital approved the study protocol.

### Assessments

Basic demographic characteristics of patients and their carers were collected. The Chinese version of the HCL-33 was used with patients, and the HCL-33-EA was used with their carers. The HCL-33 (9) and HCL-33-EA (10) are self-rated questionnaires on patients' hypomanic symptoms. The total scores of the two scales are calculated by adding up all the positive answers and the total score ranges from 0 to 33 (9, 10). The Chinese versions of the two scales have been validated previously (9, 19).

### Statistical Analyses

The sample size was calculated using G*power (27). Using reported screening abilities of HCL scales (9, 12) and the allocation ratio of MDD and BD patients as reported previously (4, 5) and given the alpha error probability of 0.05, and a conservative medium effect size of 0.5, at least 294 participants, with 68 BD and 226 MDD patients, would be needed.

SPSS 25 (IBM Corp, Armonk) was used for all analyses. The HCL-33 and the HCL-33-EA were compared by sensitivity, specificity, PPV and NPV. The Receiver Operating Characteristic (ROC) curve was plotted to represent the ability of the instrument to distinguish between BD and MDD. The internal consistency was measured by the Cronbach's alpha, in which excellent α coefficient was defined as ≥0.90, good was defined as 0.80–0.89, and adequate was defined as 0.70–0.79 (28). Following previous studies (12, 29), the optimal cut-off was calculated using the maximum sum score of sensitivity + specificity – 1, according to Youden's index (30). Cohen's kappa was used to determine the consistency between HCL-33 and the HCL-33-EA, with below 0.40 as poor agreement, 0.40–0.75 as fair to good agreement, and above 0.75 as excellent agreement (31). Statistical significance was set at was set at <0.05 (two-tailed).

## Results

A total number of 269 patients (MDD: *n* = 185 and BD: *n* = 84), and 269 carers who met the study criteria were included during the study period for analyses. Their basic characteristics are shown in Table 1.

**Table 1:** Characteristics of patients with mood disorders and their carers.

	**Carers (*****n*** **= 269)**		**Patients (*****n*** **= 269)**			
			**MDD (*****n*** **= 185)**		**BD (*****n*** **= 84)**	
	***N***	**%**	***N***	**%**	***N***	**%**
Men	149	55.4	42	22.7	22	26.2
Married	236	87.7	113	61.1	37	44.0
Employed	266	98.9	164	88.6	73	86.9
	**Mean**	**SD**	**Mean**	**SD**	**Mean**	**SD**
Age (years)	42.7	11.7	35.9	12.8	32.9	12.4
Education (years)	13.1	2.9	13.9	3.1	14.1	2.8

*BD, bipolar disorder; MDD, major depressive disorder*.

The Cronbach's alpha of the HCL-33 was 0.867 and that the HCL-33-EA was 0.872, which suggests that the two scales had good reliability, while the HCL-33-EA had slightly higher reliability than the HCL-33. The mean sum score of the HCL-33 and the HCL-33-EA were 15.3 (SD = 6.5) and 11.0 (SD = 6.2), respectively. Using the optimal cut-offs calculated in the current sample, there was a significant, but poor agreement between the HCL-33 and the HCL-33-EA (*k* = 0.36, *p* < 0.001). The proportion of BD was relatively higher in the current sample than the calculated proportion, thus the chance agreement should be adjusted according to the influence of prevalence and bias (32). The Prevalence and Bias Adjusted Kappa (PBAK) was also calculated (*k* = 0.37, prevalence index = −0.22, bias index = −0.17). As shown in Table 2, the HCL-33 and HCL-33-EA was compared in terms of sensitivity, specificity, Youden's *J*, PPV, NPV, and AUC, using the optimal cut-offs of the two scales.

**Table 2:** Comparison between the HCL-33 and the HCL-33-EA in terms of sensitivity, specificity, PPV, NPV, and Area Under the Curve (AUC) for the detection of bipolar disorder from major depressive disorder.

**Scales**	**AUC**	**95% CI**	**Cut-off value**	**Sensitivity (SE)**	**Specificity (SP)**	**Youden's *J***	**PPV**	**NPV**
HCL-33	0.73	0.66–0.81	18	0.66	0.71	0.37	0.52	0.84
			19	0.62	0.78	0.39	0.57	0.83
			20^a^	0.59	0.82	0.41	0.60	0.82
			21	0.47	0.87	0.34	0.63	0.79
HCL-33-EA	0.82	0.77–0.88	9	0.89	0.55	0.43	0.45	0.91
			10	0.86	0.61	0.46	0.47	0.89
			11^a^	0.83	0.68	0.51	0.50	0.89
			12	0.75	0.74	0.49	0.53	0.86

a *Optimal cutoff in current sample; PPV, Positive Predictive Value; NPV, Negative Predictive Value; AUC, Area under the curve; CI, 95% confidence interval for AUC; HCL-33, Hypomania Checklist-33; HCL-33-EA, Hypomania Checklist-33-externl assessment*.

**Figure 1:**
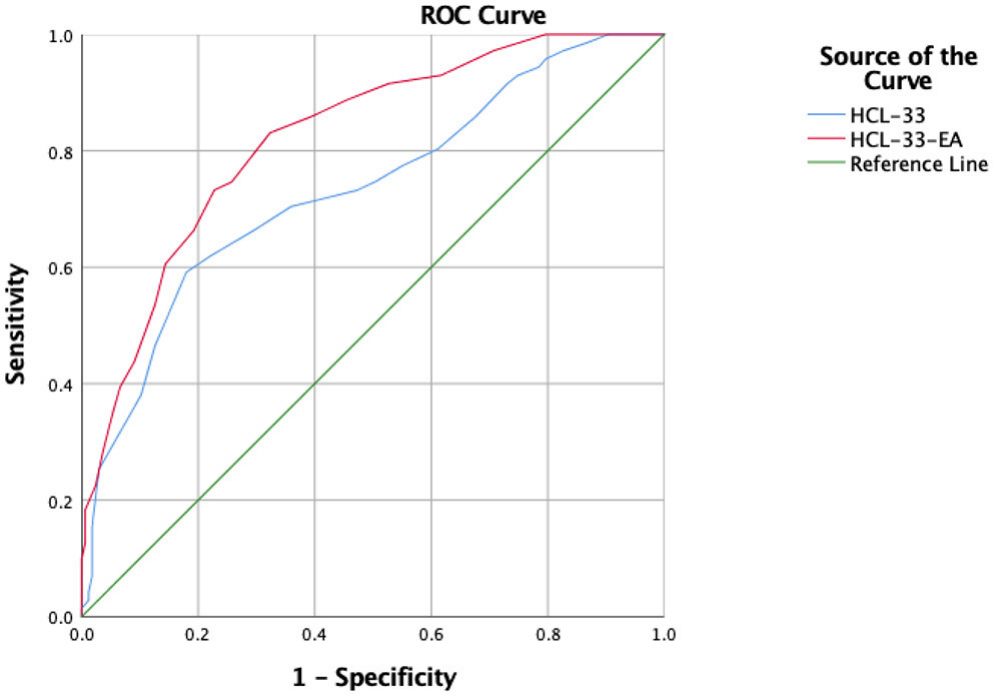
ROC curves for the HCL-33 and the HCL-33-EA. HCL-33, Hypomania Checklist-33; HCL-33-EA, Hypomania Checklist-33-external assessment.

Using Youden's index, the optimal cut-off value of the HCL-33 is 20, while the optimal cut-off value for HCL-33-EA is 11. The HCL-33-EA showed a better performance than the HCL-33 in discriminating BD from MDD (the maximum of sensitivity + specificity – 1: 0.51 vs. 0.41; Table 2). Figure 1 shows the ROC curves of the HCL-33 and the HCL-33-EA. The HCL-33-EA demonstrated better sensitivity than the HCL-33 (0.83 vs. 0.59) to correctly identify patients with BD from patients with MDD, while the HCL-33 presented better specificity than the HCL-33-EA (0.82 vs. 0.68).

## Discussion

This was the first study to compare the screening abilities of the Chinese version of the HCL-33 and the HCL-33-EA and their consistency in identifying BD from MDD. In terms of AUC, the HCL-33-EA showed better performance than the HCL-33, although the difference did not reach significance. The HCL-33-EA had higher sensitivity and the HCL-33 had higher specificity. There is no significant difference between the two scales in terms of detection BD from MDD, and thus we recommended the joint use of the two scales. Moreover, the relatively low Youden's *J* suggests that positive screenings should be confirmed in formal diagnostic interviews with a mental health professional. While a high sensitivity is the key property of a screening instrument, the relatively lower specificity indicates that false positives will likely be included in positive screenings.

Consistent with previous findings (10), this study found the HCL-33-EA was useful in assessing hypomanic symptoms. Similar to other studies (19), we found that the HCL-33-EA had higher reliability and the HCL-33-EA total score was lower than the HCL-33. This is also the first study that calculated an optimal cut-off for the HCL-33-EA. For discriminating BD from MDD, the optimal cut-off for the HCL-33-EA was 11 in the Chinese population, which is lower than the optimal cut-off of 20 for the HCL-33 in this study and 15 in a previous study (9) as expected. The discrepancy in cut-off values could be due to the different reflection and observation on hypomanic symptoms between patient's and their carer's assessments.

The HCL-33-EA had higher sensitivity than the HCL-33, which suggests that the HCL-33-EA, and carers more generally, could have better ability to correctly identify patients with hypomanic symptoms. The HCL-33 had higher specificity than the HCL-33-EA, which suggests that the HCL-33, and patients themselves, could have better ability in correctly identifying patients without hypomanic symptoms. Although the HCL-33 and the HCL-33-EA could be used according to different clinical purposes, joint use of the two scales is associated with more reliable assessment for BD patients.

The results showed a large difference in the optimal cut-off values between the HCL-33 and the HCL-33-EA, which could be partly due to the different perspective of the interviewers. For instance, a recent study found different feelings about clinical features of anxiety between patients and their carers using the pediatric short form (completed by patients) and proxy form (completed by carers) of the National Institutes of Health's Patient Reported Outcomes Measurement Information System scale. Carers tended to identify the existence of anxiety more than the patients themselves (33). In addition, recall bias may partly account for the discrepancy between mood disorder patients' and their carers' assessments (34). Compared to patients, their carers were less likely to have recall bias; therefore, the HCL-33-EA version is more prone to identify BD than the HCL-33. The results showed there were significant, but poor agreement between the HCL-33 and the HCL-33-EA assessments (*k* = 0.36, *p* < 0.001). The gap between the subjective and the external assessments indicates the importance of the combined use of the HCL-33 and the HCL-33-EA in identifying BD patients in clinical practice. Furthermore, the HCL-33-EA had a higher sensitivity, while the HCL-33 had a higher specificity in identifying BD patients from MDD patients in this study, which suggests that patients' carers were more likely to detect BD, while patients themselves were more likely to recognize the absence of BD. Hence, use of the two scales should be considered based on the assessment purpose in clinical research or daily practice (i.e., prefer sensitivity or specificity).

This study had several limitations. First, all participants were consecutively recruited in one major psychiatric hospital, and a relatively higher proportion of BD patients, therefore the findings cannot be generalized to patients in other clinical settings, which may bias the results to uncertain extent. However, the higher proportion of BD patients in the study sample reflects the real situation in daily practice in this major tertiary psychiatric hospital in China. Second, the ICD-10 is used in clinical practice in China, therefore, the diagnosis of BD-I and BD-II cannot be established. As such, we did not examine screening capacities for BD subtypes. In addition, the Mini-International Neuropsychiatric Interview Version 5.0 cannot generate DSM-5 diagnoses. Therefore, certain specifiers related to BD, such as mixed depression, could not be assessed. Third, we were unable to explore the differences in screening abilities between different carers who provided data on the HCL-33-EA (e.g., parents, spouses, or friends). Variation in the sources of self-reports should be examined in future studies. Fourth, the psychometric properties of the HCL-33-EA need to be tested with additional measures, such as the MDQ, as reference tools. Fifth, the possibility of recall bias could not be excluded, particularly in the HCL-33 assessment (34). Finally, diagnostic properties of screening instruments (e.g., sensitivity, specificity, PPV, and NPV) are associated with disease prevalence, study design, sampling method, and sample size (35). Similar to previous studies on the psychometric properties of the HCL scales (11, 21–23), the sample size of this study was relatively small; therefore, our findings are tentative, and will need to be replicated in future studies with a larger sample size and a multicentre design.

## Conclusions

In conclusion, both the HCL-33 and the HCL-33-EA appeared to have acceptable psychometric properties and screening abilities in accurately detecting and differentiating between BD and MDD. The two scales could facilitate identification of people with BD in clinical practice, and use of the two scales should be considered based on the assessment purpose in clinical research or daily practice (i.e., prefer sensitivity or specificity).

## Data Availability Statement

The Ethics Committee of Beijing Anding Hospital that approved the study prohibits the authors from making the research data set publicly available. Readers and all interested researchers may contact Dr. Gang Wang (Email address: gangwangdoc@gmail.com) for details. Dr. Wang could apply to the Ethics Committee of Beijing Anding Hospital for the release of the data.

## Ethics Statement

The studies involving human participants were reviewed and approved by Ethics Committee of Beijing Anding Hospital. The patients/participants provided their written informed consent to participate in this study. Written informed consent for publication was obtained.

## Author Contributions

Y-YW, GW, and Y-TX: study design. MF, CG, Y-YW, and YF: analysis and interpretation of data. Y-YW, GW, and Y-TX: drafting of the manuscript. BH and GU: critical revision of the manuscript. All authors approval of the final version for publication.

## Conflict of Interest

The authors declare that the research was conducted in the absence of any commercial or financial relationships that could be construed as a potential conflict of interest.

## Abbreviations

HCL-33: 33-item Hypomania Checklist (HCL-33)
HCL-33-EA: the 33-item Hypomania Checklist External Assessment.
